# Evaluation of Serum Levels of Matrix MetalloProteinase-9 (MMP-9) in Oral Squamous Cell Carcinoma and Its Clinicopathological Correlation

**DOI:** 10.7759/cureus.34954

**Published:** 2023-02-14

**Authors:** Georgia Benitha, Pratibha Ramani, Selvaraj Jayaraman, Abilasha R, Karthikeyan Ramalingam, Murugesan Krishnan

**Affiliations:** 1 Oral Pathology, Saveetha Dental College and Hospitals, Saveetha Institute of Medical and Technical Sciences, Chennai, IND; 2 Biochemistry and Molecular Biology, Saveetha Dental College and Hospitals, Saveetha Institute of Medical and Technical Sciences, Chennai, IND; 3 Oral and Maxillofacial Surgery, Saveetha Dental College and Hospitals, Saveetha Institute of Medical and Technical Sciences, Chennai, IND

**Keywords:** prediction, nodal involvement, tumor staging, oral squamous cell carcinoma, oral cancer, mmp-9, serum levels

## Abstract

Background: One of the most prevalent malignancies in India is oral squamous cell carcinoma (OSCC), which is found in more than 90% of cancer cases and has a reduced survival rate of 30%. Matrix metalloproteinases (MMPs) are zinc-containing and calcium-dependent endopeptidases that regulate angiogenesis, migration, and proliferation. MMP-9 in OSCC increases tumor progression through angiogenesis, degrades the basement membrane, and facilitates metastasis by changes in tissue shape. Its overexpression in OSCC has also been shown to have prognostic significance.

Aim: This study aims to evaluate the serum levels of MMP-9 in OSCC patients and healthy controls and to correlate with its clinicopathological staging.

Materials and methods: This study included 40 individuals; 20 patients with OSCC and 20 healthy controls. MMP-9 was determined in serum samples utilizing enzyme-linked immunosorbent assays.

Results: Descriptive statistics showed that 90% of the patients included in the OSCC groups were above 40 years, and 85% were males. There was a significant increase in the serum level of MMP-9 in OSCC patients compared to healthy controls with a mean difference of +28% (393.21 pg/ml) and a significant p-value of 0.001. (1365.80 ±236.414 pg/ml vs 973.67 ± 83.416 pg/ml). There was a significant increase in the serum levels of MMP-9 among the tumor stages and nodal involvement with a significant p-value of 0.002 and 0.001. No significant association was found between the age and gender groups in OSCC patients and serum levels of MMP-9.

Conclusion: MMP-9 was significantly increased in OSCC when compared to healthy controls. Hence, MMP-9 can be used as a prognostic indicator in assessing tumor staging and nodal involvement.

## Introduction

Matrix metalloproteinase (MMP) is a zinc-dependent endopeptidase that involves the degradation of collagen in the extracellular matrix. They play an important role in tissue repair and extracellular matrix (ECM) remodeling, which in turn promotes cancer invasion [1,2]. It is widely known that the MMP-9 member of the MMP family, which has collagenase and gelatinase activity, is strongly expressed at the invasive front of OSCC and correlated to the progression and aggressiveness of OSCC. The secretion of substances like transforming growth factor-beta and the vascular endothelial growth factor is stimulated by MMP-9, which promotes angiogenesis and tumor growth [3,4].

Recent literature provides well-established evidence regarding the overexpression of MMP-9 in cancer [5,6]. Additionally, it has been reported that MMP-9 polymorphism is associated with a greater risk of developing oral cancer [7]. MMP-9 has been shown to contribute to the pathogenesis of cancer by destroying type IV collagen, elastin, and fibronectin as well as by regulating angiogenesis [8,9].

Among the cancers reported, oral cancer is the 6th most common type. Oral squamous cell carcinomas (OSCC) are the most common type of oral cancer, accounting for more than 90% of all cases [10,11]. Incidence rates for oral cancer, which account for nearly 5% of all malignant tumors worldwide, are 8.2 per 100,000 for men and 2.8 per 100,000 for women annually. Additionally, the fact that 60 to 80% of patients with stages I-II of the disease survive indicates that early detection of oral cancer in high-risk patients has a high chance of being successfully treated [12]. Only 15% of the patients had their disease locally diagnosed. However, the prognosis is worse for OSCC patients who have malignant progression, such as distant metastases [12]. The local invasion and dissemination to the lymph nodes are responsible for the disease's poor prognosis (recurrence and metastasis) [12]. Therefore, to give targeted therapy, it is necessary to discover efficient biomarkers to predict tumor development and prognosis.

Molecular and biochemical alterations might provide essential information for predicting tumor behavior, recurrence, and metastatic potential even when the surrounding healthy tissues of oral cancers may appear clinically normal (concept of field cancerization) [5,6]. ECM remodeling is a crucial factor in determining the behavior and development of cancer because it makes it easier for cancer cells in the tumor microenvironment to invade nearby tissue. MMP-9 is an important marker that plays an important role in extracellular matrix degradation. One of the main constituents of the basement membrane, type IV collagen, is degraded by MMP9 (BM) [13,14]. Although considerable evidence has accumulated showing overexpression of MMP-9 in OSCC tissue, increased MMP9 protein expression has been associated with distant, nodal, and recurrent metastasis [15].

Recently, the emphasis has been on finding molecular markers in human fluids including serum, saliva, and urine to diagnose the condition, determine its prognosis, and predict its progression [16]. Since blood-based testing is simple, inexpensive, noninvasive, and allows for repeated samples, it is preferred over other types of testing for patient screening and monitoring. MMP-9 is secreted into the blood through the extracellular matrix.

Although various studies have been done to assess the immunohistochemical and gene expression of MMP9, few studies have reported the serum levels of MMP9 in OSCC in the Iranian population. Studies evaluating the serum levels of MMP-9 in OSCC patients in the Indian population are sparse [15].

This study aims to evaluate the serum level of MMP9 in OSCC patients when compared to the healthy controls and to correlate with the clinicopathological findings. 

## Materials and methods

Study design

This was a cross-sectional study conducted in the Department of Oral Pathology after getting ethical clearance from Saveetha Dental College Institutional Human Ethics Committee (approval no: IHEC/SDC/PhD/OPATH-2212/22/001). Following an explanation of the study and information about the sampling procedures, each subject signed a written informed consent form.

Inclusion criteria

Patients were diagnosed with OSCC based on oral clinical examination and confirmed by histopathological examination. The controls were generally healthy people without any history of tobacco smoking or drinking alcohol and without any underlying systemic diseases, any cancer, or inflammatory oral lesions.

Exclusion criteria

Patients with autoimmune disorders, hepatitis, inflammatory oral lesions, periodontal disease, systemic disease, prior malignancy or human immunodeficiency virus infection, as well as pregnant or breastfeeding women, were excluded from the study.

Sample collection

**Figure 1:**
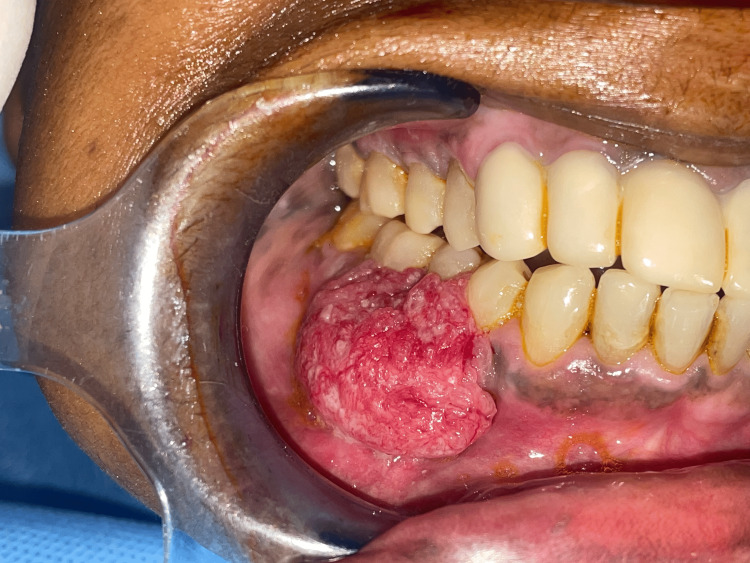
Clinical picture Clinical picture showing an ulcero-proliferative growth involving the gingivo-buccal region

The sample size of 40 for our study was calculated utilising Gpower and epidemiological data of our region. Hence, 20 patients with OSCC were in the study group and 20 healthy individuals were included as controls. The controls were age-matched and gender-matched to the study group. After obtaining written consent, serum samples were collected from patients in the OSCC group and the control group (Figure 1).

**Figure 2:**
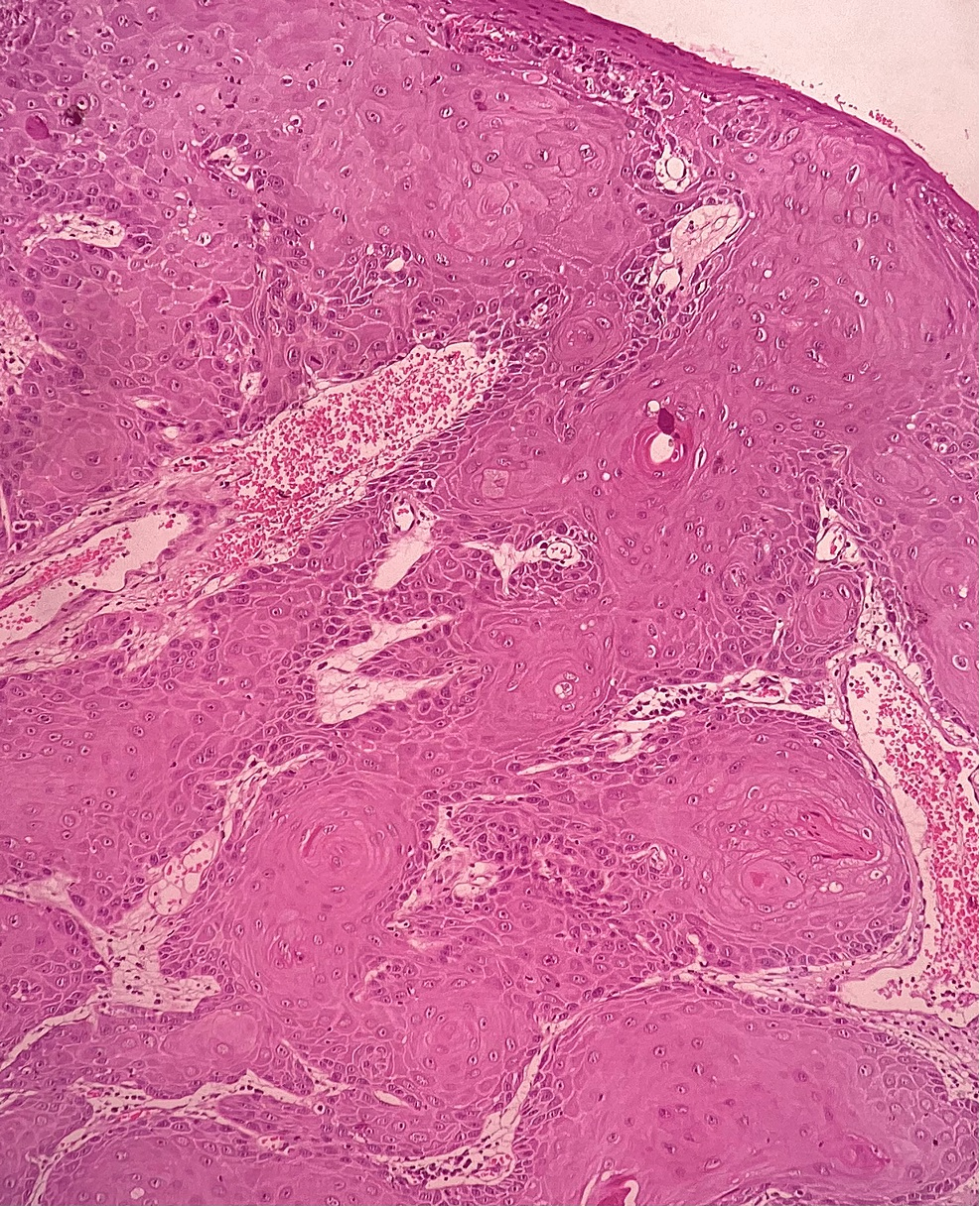
Histopathology Photomicrograph depicting malignant epithelial islands within the connective tissue (H&E 20x)

The demographic details for all study participants were collected. The clinicopathological characteristics of tumors in patients with OSCC were recorded. Age, gender, tumor site, tumor stage as determined by the American Joint Committee on Cancer (AJCC) TNM classification, and histological tumor grade were collected (Figure 2).

Procedure

Four milliliters (ml) of fasting venous blood was drawn under stringent aseptic conditions from the cubital vein of study participants after they signed an informed consent form. Blood was drawn and placed in sterile tubes and vacutainers. It was allowed to be centrifuged for 10 minutes at 3000 rpm to separate the serum after two hours at room temperature. Until the levels of MMP-9 were measured, the serum samples were stored at 20°C.

**Figure 3:**
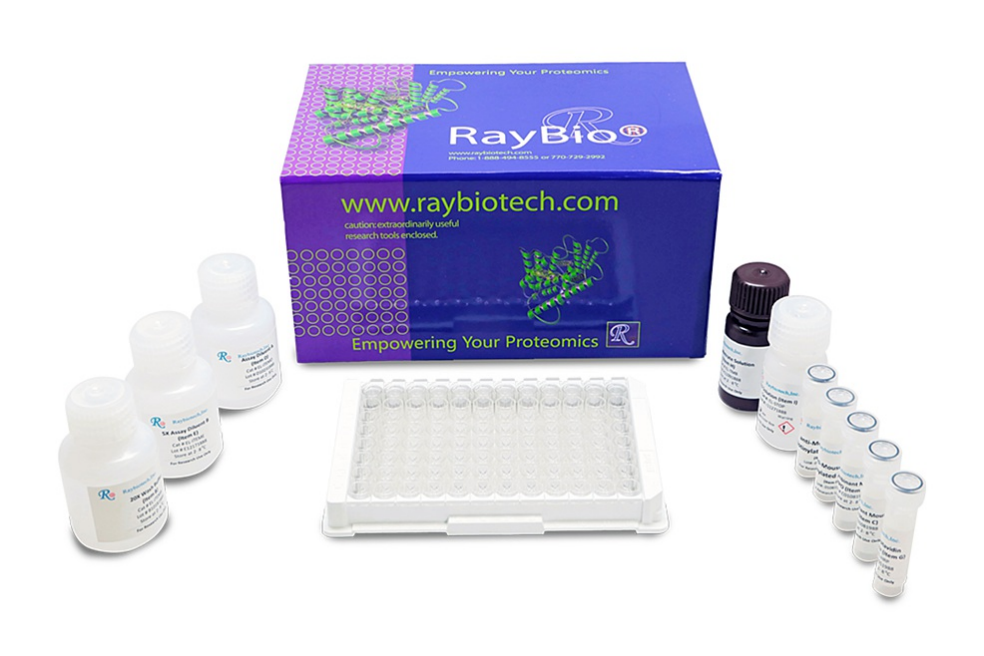
Picture of Raybiotech ELISA kit for MMP-9 Picture showing the RayBio® Human MMP-9 ELISA Kit for cell culture supernatants, heparin treated plasma, and serum samples. ELISA: enzyme-linked immunosorbent assay; MMP-9: matrix metalloproteinase-9

The serum MMP-9 concentrations were measured using RayBioTech Human MMP-9 enzyme-linked immunosorbent assay (ELISA) kit (RayBioTech Life Inc, Georgia, USA) (Figure 3).

The manufacturer's protocol was followed for analysis.

Every assay was carried out twice. The kit used a sandwich ELISA method, in which the sample's MMP-9 binds to a polyclonal antibody that is specific to MMP-9 and is coated on a microplate. MMP-9 that has been attached to the plate is detected by a secondary, biotinylated MMP-9-specific antibody. At 450 nm, the intensity was measured. The final readings were calculated in pg/ml and a set of standards was loaded alongside. A trained and calibrated professional carried out the procedure.

Statistical analysis

The serum levels of MMP-9 of our study were assessed using SPSS version 19 (IBM Corp., Armonk, NY) with a chi-square test and one-way ANOVA The correlation between MMP-9 levels and clinicopathological findings in patients with OSCC was assessed using Pearson's correlation. P value < 0.05 was considered to be statistically significant.

## Results

This study included a total of 40 samples of which there were 20 patients with OSCC and 20 healthy controls. About 90% of the patients included in the OSCC groups were above 40 years, and nearly 85% were male. The control group included 50% of male patients and 50% of female patients. Among the SCC cases, 40% were in the buccal mucosa region, 30% in the gingivobuccal sulcus region, and 30% in the tongue (Table 1).

**Table 1:** Comparison of mean serum MMP-9 levels Table represents the mean serum value of MMP-9 in subgroups of OSCC patients. There was a significant p-value associated with histological grade, tumor size, staging, and nodal involvement (p-value < 0.05). MMP-9: matrix metalloproteinase-9; OSCC: oral squamous cell carcinoma

PARAMETERS	N =20	FREQUENCY	Mean ± SD Serum MMP9 (pg/ml)	P value
AGE				
< 40 years	2	10%	1173.32 ± 213.22	0.06
>40 years	18	90%	1543.87± 213.21	
GENDER				
MALE	17	85%	1653.21± 118.21	0.08
FEMALE	3	15%	1585.42±323.22	
HISTOLOGICAL GRADE				
WDSCC (Well differentiated squamous cell carcinoma)	7	35%	1321.12± 125.21	0.001*
MDSCC (Moderately differentiated squamous cell carcinoma)	11	55%	1585.54± 236.43	
PDSCC (Poorly differentiated squamous cell carcinoma)	2	10%	1763.43± 386.32	
TUMOR SITE				
Buccal mucosa	8	40%	1682.39± 384.21	0.002*
Gingivobuccal sulcus	6	30%	1481.39± 326.11	
Tongue	6	30%	1029.23±212.22	
Tumor size				
2-4 cM(T2)	12	60%	1328.32± 433.33	0.001*
>4cm (T3)	8	40%	1618.93±172.86	
Nodal involvement				
N0	6	30%	1029.28±.302.22	0.001*
N1	7	35%	1376.28±212.99	
N2	7	35%	1549.39±273.22	

Considering the TNM classification, 60% of cases were in the T2 grade, and 40% of cases were in the T3 grade. Lymph node involvement was observed in 70% of the included cases with 35% in N1 and 35% in N2. There was no metastasis. 

**Figure 4:**
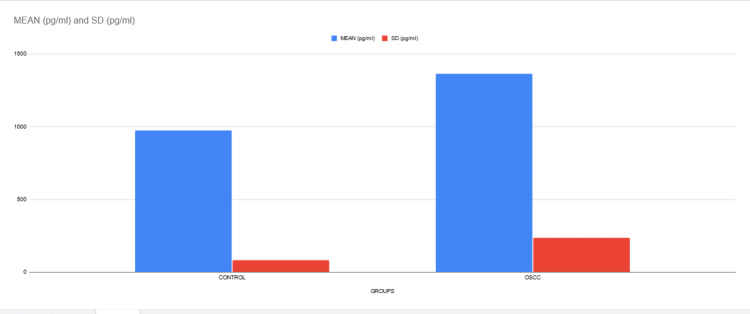
Graph Graphical representation of mean and SD of MMP levels among the study group and control group. SD: standard deviation; MMP: matrix metalloproteinase

The OSCC group has a higher level of serum MMP-9 when compared to healthy controls, with a mean difference of +28%(393.21) and a significant p-value of 0.001 (1365.80 ±236.414 VS 973.67 ± 83.416) (Table 2) (Figure 4).

**Table 2:** Serum levels of MMP-9 Table indicates the serum level of MMP-9 in the OSCC group vs healthy controls with a significant p-value (0.001). MMP-9: matrix metalloproteinase-9; OSCC: oral squamous cell carcinoma

GROUPS	MEAN ± SD (pg/ml)	P VALUE
CONTROL	973.67 ± 83.416	0.001*
OSCC	1365.80 ±236.414	

When compared between the subgroups of OSCC, the serum level of MMP-9 was higher in the buccal mucosa region compared to the tongue and gingivo-buccal sulcus regions with no statistically significant p-value. (1682.39± 384.21 vs 1481.39± 326.11,1029.23±212.22). There was no significant difference in the serum level of MMP-9 in male and female patients. (p-value > 0.05).

Serum levels of MMP-9 increased statistically in T2 when compared to T1 (1618.93±172.86 vs 1328.32± 433.33) with a p-value of 0.002. There was a gradual increase in the serum levels of MMP-9 from N0 to N2 (1549.39±273.22,1376.28±212.99, 1029.28±.302.22) with a significant p-value of 0.001. 

## Discussion

In India, oral cancer is a major public health issue. Globocan reported that 13,24,413 new cases and 8,51,678 deaths were reported in India in 2020 [17]. OSCC can rapidly spread and invade nearby tissue. It has a notable propensity to invade the regional lymph nodes, most likely very early in carcinogenesis [18].

The metastatic potential of OSCC depends on its ability to degrade ECM, penetrate the basement membrane, initiate tumor angiogenesis, and infiltrate surrounding tissues and blood vessels [18]. MMPs are important for the degradation of ECM and the basement membrane. Numerous clinical and experimental studies have associated an increase in various MMPs particularly MMP-2 and MMP-9 with cancer development. MMP-9 is a type IV collagenase that can degrade the extracellular membrane and basement membrane’s primary structural proteins [19]. Numerous studies have shown that increased expression of these MMPs leads to tumor progression and metastasis [20]. Previous studies also reported that the expression of MMP-9 is associated with the aggressiveness of the OSCC [3]. Hence, our study evaluated the serum levels of MMP-9 in OSCC tissue samples. 

According to the results of this study, there is a mean difference of (+28%) in the serum levels of MMP-9 between OSCC and healthy controls. This was in accordance with previous research by Shpitzer et al. who found that MMP-9 increased in OSCC patients by 35% to 39% compared to controls [21].

We found no significant association between the age and gender of OSCC patients with serum MMP-9 levels. This was in accordance with previous studies examining serum levels of MMPs in breast ductal carcinoma and salivary gland tumor, with no association between age and gender [22]. However, de Vicente et al. showed contradictory results with high levels found in patients under 60 years [23]. This controversy in literature can be due to the diversity in the study samples which makes it difficult to evaluate the MMPs and their exact role in different gender and age groups.

We observed that an increase in serum level of MMP-9 significantly correlated with the increase in T grade and lymph node involvement. Similar to our findings, some previous studies have shown a significant correlation between MMP-9 with lymph node involvement [24]. Previous literature by Kato et al. demonstrated that MMP-9 expression was high in OSCC and was associated with advanced stages of the disease [25,26]. Ikebe et al. concluded that the OSCC tumor's gelatinolytic activity and elevated expression of MMP-2 and MMP-9 were associated with the tumor's invasiveness but not with the metastatic potential of the OSCC tumor [27]. However, many researchers have presented contradictory results. Katayama et al. reported no significant correlation between MMP-9 expression and the tumor stage in OSCC patients [27]. Our results were contradictory to previous literature by de Vicente et al. [23] which concluded that MMP-9 expression had no significant association with the clinical variables, such as tumor stage or recurrence rate. 

MMP-9 has an important role in ECM remodeling, metastasis, angiogenesis, apoptosis, and cancer progression [28]. It has been determined that MMP-9 encourages metastasis by causing the ECM, a physical barrier, to break down and by induction of SNAIL (transcription factor), which initiates the epithelial-mesenchymal transition and changes the morphology and reduces the cell adhesion molecules, allowing the carcinoma cells to migrate [28,29]. Due to the disruption of the basement membrane, the endothelial cell migrates from the existing vessel to produce a new blood vessel and also releases ECM-bound factor. Anti-angiogenic factors are produced by MMP-9 from their precursors to inhibit angiogenesis, and vascular endothelial growth factor (VEGF) is produced and activated from extracellular proteoglycans to promote angiogenesis [29].

Hence, the MMP-9 enzyme and its inhibitors may be essential targets for anticancer therapies. Potential anticancer effects have been related to the selective inhibition of certain enzymes at specific sites [29]. Depending on the structural studies, many MMP-9 inhibitors have been designed to be used in the treatment of cancer.

Limitation

In this study, we measured the concentration of human MMP-9 in both the control and OSCC samples using a commercially available human MMP-9 sandwich ELISA kit procured from RayBioTech Human MMP-9 ELISA kit (Catalog no. ELH-MMP-9) as per manufacturer’s instructions. Since we have measured MMP-9 level in 40 patients (n=40), more number of samples are required to find the exact cut-off value for the same, and studies are under progress.

However, the concentration of MMP-9 in both the control and OSCC patient samples was within the standard range of 8.23 pg/mL-6000 pg/mL and the concentration of MMP-9 in all the patient samples ranged from 136-073 pg/ml.

Although only serum expression was examined in our study, future investigations of MMP-9 levels in salivary samples could be performed. The MMP-9 levels in serum with their correlation to patients' survival and response to therapy will provide valuable information.

## Conclusions

In conclusion, patients with OSCC had significantly higher serum levels of MMP-9 than healthy patients. MMP-9 could be a superior marker for determining lymph node involvement and tumor grade. Within the limits of this clinical study, serum MMP-9 has a predictive role in the diagnosis of OSCC. This study evaluated the association between MMP-9 serum levels and tumor metastasis, staging, and nodal involvement. This will provide insight into the function of MMP-9 as a significant adjuvant serological biomarker in predicting disease progression. These findings contribute to the essential involvement of MMP-9 in carcinogenesis and its potential value in aiding surgeons identify high-risk individuals who may need more aggressive therapy and close monitoring.
